# A Case Report of Acute Necrotizing Encephalitis

**DOI:** 10.7759/cureus.21100

**Published:** 2022-01-11

**Authors:** Khalilalrahman Alshantti, Chandran Nadarajan, Mitchell Modi Mijol

**Affiliations:** 1 Radiology, Universiti Sains Malaysia Hospital, Kubang Kerian, MYS

**Keywords:** neuroradiology, paediatric radiology, childhood encephalitis, acute hemorrhagic necrotizing encephalitis, acute necrotizing encephalitis

## Abstract

Acute necrotizing encephalitis (ANEC) is a rare entity seen primarily in East Asian infants and previously healthy children. A 5-year-old boy complained of fever and seizures, which developed into status epilepticus. Computed Tomography (CT) and Magnetic Resonance Imaging (MRI) brains showed acute necrotizing encephalitis features. Empirical treatment for meningoencephalitis with supportive therapy was administered. MRI was then repeated 25 days post-therapy, which showed the previously seen abnormal signal intensities resolution. The patient was subsequently discharged home with moderate neurological impairment. Although ANEC is a rare disease, a typical clinical scenario and MRI findings should prompt recognition of the disease, essential for treatment.

## Introduction

Acute necrotizing encephalopathy (ANEC) is an atypical encephalopathy seen only in previously healthy infants and children. Initially, it was thought to occur only in Japan and Taiwan [1]. However, sporadic cases worldwide have been reported in the literature [2]. The etiology and actual causative pathogen have yet to be discovered. Mycoplasma, herpes simplex virus, human herpesvirus-6, and influenza virus are the primary pathogens implicated in a patient's clinical deterioration [3]. Even though these pathogens have been identified as the joint causal agent, it is currently thought that the illness is most likely immunological or metabolic [2]. It has been hypothesized that cytokines, including tumor necrosis factor receptor-1, interleukin-1, and interleukin-6, may affect the disease's progression [4]. The development of seizures, altered consciousness, vomiting, and different hepatic failure characterized the acute necrotizing encephalopathy course. Some of the MRI findings of ANEC include multiple symmetrical lesions in the thalami, cerebellar white matter, brainstem tegmentum, periventricular white matter, and putamina [5].

## Case presentation

A healthy 5-year-old boy with no previous comorbidities presented with high-grade fever(39°C) for two days with more than five episodes of generalized tonic-clonic seizures followed it. Each seizures episode lasted for about one minute. Post-ictal, he appeared lethargic. The child was then brought to the hospital immediately by the parents. In the emergency department, he developed status epilepticus, which lasted for over an hour, and aborted after administering valium suppository 5mg, IV Valium 2mg, and IV Phenytoin 400mg over 30 minutes. His parents deny any history of abnormal behavior, upper respiratory tract infection, or gastrointestinal symptoms before the beginning of symptoms. There was no family history of seizures or neurological disorders.

**Figure 1:**
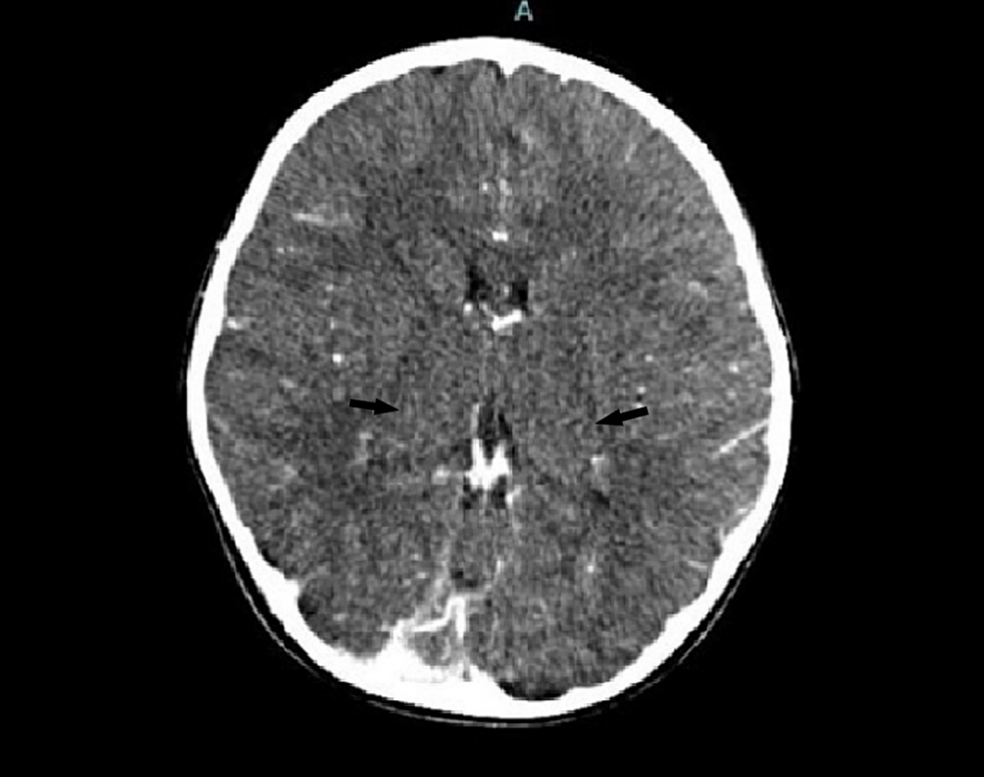
Contrasted CT brain shows bulky bilateral thalami. No focal lesion. No leptomeningeal enhancement.

**Figure 2:**
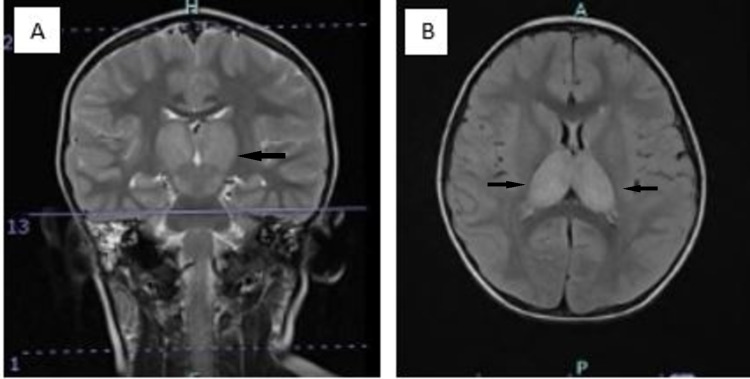
Coronal T2 (A) shows abnormal signal intensities in bilateral thalami. No other abnormal signal intensities. Axial FLAIR (B) sequence shows a non-suppressed hyperintense signal in both thalami.

**Figure 3:**
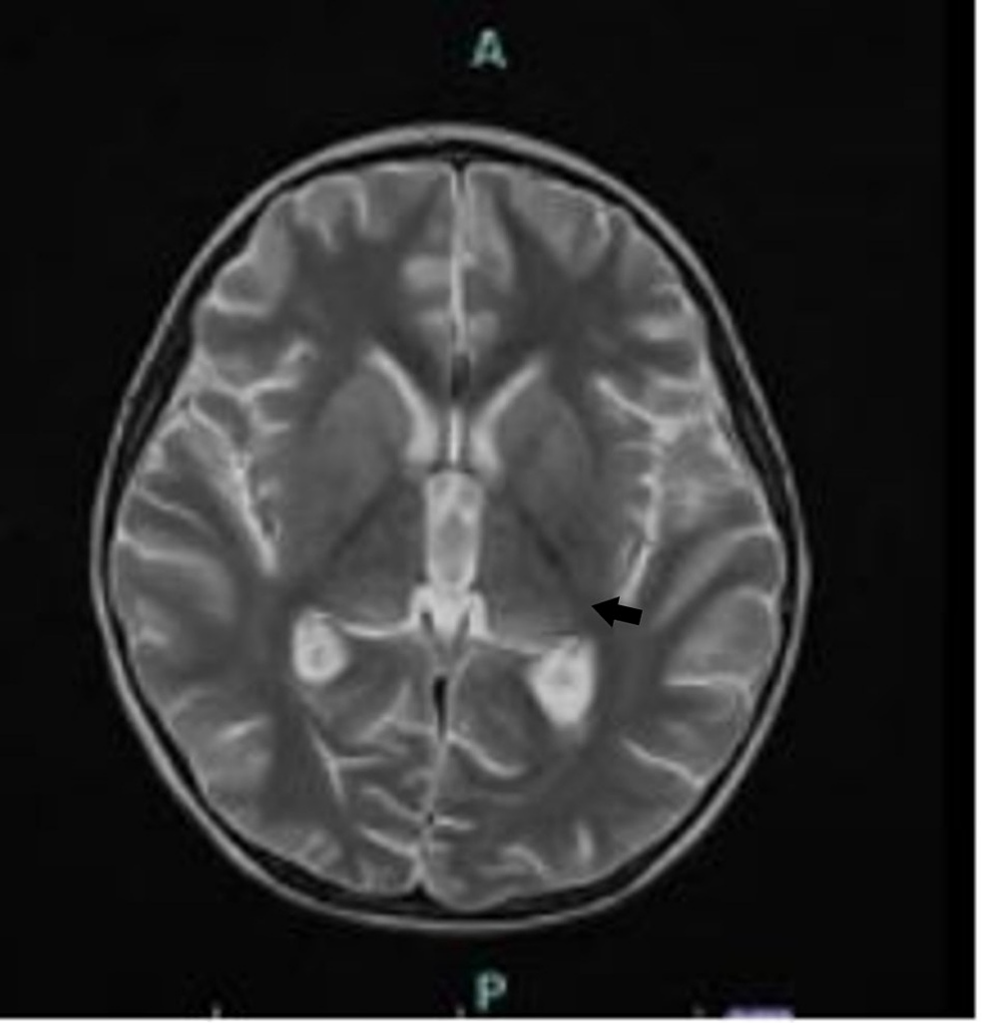
Axial T2 25 days later shows resolution of MRI changes, Normal appearance of the thalamus (arrow).

Complete blood count performed upon arrival showed leukocytosis TW 15.3. Other parameters, such as venous blood gas (VBG), oxygenation level, and blood pressure, were normal. CSF study was requested. However, the child's parents were not keen on lumbar puncture. Neurological examination showed upper motor neuron signs in all four limbs with severe hypertonia. The rest of the systems' examinations were unremarkable. The child underwent elective intubation for airway protection. The patient was treated empirically for meningoencephalitis, covered by intravenous (IV) ceftriaxone and Acyclovir for 14 days. Another supported treatment was prescribed, such as IV immunoglobulin for five courses over three months, oral prednisolone 30mg, and IV methylprednisolone 200mg for two courses. Each course lasted for three days. The patient was discharged home with moderate neurological impairment. He had spasticity of all four limbs. An emergency contrasted CT brain was done, showing enlarged bilateral thalamus with no CT evidence of meningeal enhancement, suggesting meningitis (Figure 1). Spinal cerebrospinal fluid (CSF) study results were unremarkable. MRI brain was performed three days later, which showed bilateral symmetrical abnormal thalamic intensities, which appear hyperintense on T2 and FLAIR sequence (Figure 2). No other abnormal signal intensities in the rest of the brain parenchyma. MRI brain repeated 25 days later, post-treatment showed resolution of abnormal signal intensities (Figure 3).

 

## Discussion

The acute necrotizing encephalopathy of childhood is rare, with over 110 cases reported in the literature [6]. Our patient presented with high-grade fever, multiple seizures episodes, and neurological disturbances. In ANEC, the symptoms and signs are nonspecific. According to Mizuguchi et al., 40% of the patients had convulsions, 28% had decreased consciousness, and 20% had vomiting [1]. An increase in CSF protein has been found in a CSF study. However, no aberrant cells were detected [7]. In the chronic stage, motor impairments like intention tremor, ataxia, speech difficulty, choreoathetosis, and spasticity are often developed. Focal neurologic symptoms, such as hemiparesis and abnormal extraocular motility, could also be present. No specific changes are seen in the laboratory tests in the patient with ANEC, except for a rise in CSF protein without pleocytosis and elevated liver enzymes [3]. However, that was not seen in our patient. This encephalopathy presents as multifocal, symmetrical brain lesions. It affects the thalamus or the white matter at the periventricular region, cerebellar medulla, or brainstem tegmentum. The changes can be seen using ultrasonography, CT, and MRI of the brain [6]. Disorders that affect the deep grey matter are common differentials, such as toxic encephalopathy, hemolytic uraemic syndrome, hemorrhagic shock, and encephalopathy syndrome. Metabolic disorders are ruled out based on the biochemical parameters and clinical findings. The main differentials for ANEC would be acute disseminated encephalomyelitis (ADEM) and Reye's disease. In ADEM, white matter and grey matter involvements are typically bilateral but asymmetrical. In Reye's disease, a history of aspirin consumption post-viral infection with liver and renal dysfunction may be noted [8]. In our case, the management was symptomatic and supportive, with hydration, corticosteroids, and anticonvulsants. Initially, antiviral drugs were prescribed and halted once the viral serology showed no evidence of herpes simplex viral infection.

## Conclusions

Here we shared a case of ANEC with no specific clinical presentation or treatment, which was diagnosed based on its hallmark imaging features, which are diagnostically significant. Hence, the radiologist plays a significant role in this diagnosis since it is a rare disease with no specific clinical features. The diagnosis can be made based on its characteristic radiological features. Despite the rarity of this disease, the radiologist should know these features of this disease as it is essential to recognize it early for prompt treatment and prevent unnecessary surgical intervention. Finally, our patient received supportive treatment and was discharged well.
